# Medical Education

**Published:** 1858-03

**Authors:** 


### EDITORIAL.

### MEDICAL EDUCATION.

At the last annual meeting of the American Medical Association, a special committee was appointed, with instructions to report, at the coming meeting in May next, a more complete and judicious plan of instruction to be adopted by the medical colleges of our country. This is a subject of much importance, and we had hoped to see a free interchange of views concerning it through the medical journals, before it should be again brought up for formal action in the Association.

For many years past, it has been almost universally acknowledged, that the system of instruction usually pursued by the medical colleges of our country is very defective.

1st. The usual term of *four months* is too short to allow of more than a partial course on any one branch, if all the important departments of medical science are embraced in the curriculum.

2d. The system permits no natural order or succession of topics for study on the part of the student, but compels him to listen to the same incomplete course on all the branches at each successive college term.

Hence, the student who has scarcely proceeded far enough in his medical studies to become familiar with the long bones of the skeleton, is placed in the same class, and made to listen to the same details in practical medicine, surgery and obstetrics, with him who had already studied diligently three or four years.

3d. The anxiety of each faculty to include as much as possible of the various departments of medical science in the college term, has caused the number of lectures each day to be so great, as to leave a very inadequate amount of time for reading, dissections, and clinical study.

To remedy or mitigate these defects, the American Medical Association, in the early part of its history, strongly recommended the extension of the college terms to *six* months. A little reflection, however, will satisfy every intelligent physician that a *simple* extension of the lecture term would constitute only a very imperfect remedy. If the additional time was used for extending the several courses of lectures, it would leave the second and third evils wholly unmitigated. On the other hand, if the number of lectures per day were reduced in proportion to the increased length of term, so as to remedy the third evil, the first and second would remain in full force. To effect a real reform in medical college instruction, to realize a practical avoidance of the evils and defects of the present system, four things are necessary, viz:

1st. Such a revision of the several departments or chairs as will permit them to be divided into two groups—the first embracing all those branches usually considered elementary, and the second, all those more directly practical.

2d. Such an extension of the collegiate year as will allow two full lecture terms; the first, for the elementary group of branches; and the second, for the practical—thus enabling the student in attending two terms to hear, instead of a *repetition* of one imperfect course, a full review of all the departments in their natural order of succession.

3d. Such a limit to the number of lectures per day as will permit each student to give adequate time to dissections and clinical instruction.

4th. Access to the wards of a well regulated hospital must constitute a necessary appendage to every medical college; and attendance on, at least, one course of clinical instruction a requisite for graduation.

To carry these views into successful and full practical operation, we would suggest the following definite plan of college organization, and ask for it the thoughtful consideration, not only of the special committee of the American Medical Association, but of the whole profession:

1st. Make the full collegiate year embrace a regular course of instruction in the following departments, viz:

1*st.* Descriptive anatomy.
2*d.* Elementary and inorganic chemistry.
3*d.* Human histology and physiology.
4*th.* Materia medica.
5*th.* General pathology and public hygiene.
6*th.* Regional and surgical anatomy.
7*th.* Organic chemistry and toxicology.
8*th.* Practical surgery.
9*th.* Practical medicine.
10*th.* Obstetrics and diseases of women.

We would group the first five of these departments together to constitute an elementary course or term, and the last five to constitute the practical course.

2d. Let each collegiate year be divided into two terms; one called the *elementary*, and the other the *practical term.* The first may commence on the first Monday in March, and continue until the last Wednesday in June. Besides embracing a full course of lectures on each of the five branches named, it should present ample facilities for the practical study of anatomy by dissections, and a course of clinical instruction in the hospitals, devoted almost exclusively to medical and surgical pathology and diagnosis.

The second term should commence on the first Monday in October, and continue until the third Wednesday in February, at which time the annual commencement for conferring degrees should be held. This term should embrace, in addition to the full course of lectures on the five branches assigned to it, also abundant material for dissections, and a full hospital clinical course devoted more directly to the details of practical medicine and surgery.

3d. No student should be eligible to graduation until he had studied medicine and surgery three years, and attended, at least, one full collegiate year, embracing the two terms just described in their proper order. But instead of attending both college terms the same year, each student should be encouraged to attend the elementary term during the first eighteen months of study, and the practical term at the end of the second eighteen months. Of course, such students as possessed the requisite means could attend a repetition of one or both terms each year during their studies.

Such is a brief statement of the system of college instruction which we would propose; and we are confident that its adoption by all the leading colleges of the country would very speedily result in great practical benefit to the profession. It would immediately render the study of medicine more systematic or methodical, by confining the attention of the student during the first half of his studies to a smaller number of branches, and those wholly of an elementary character, but essential to qualify him to advance understandingly to the second series during the last half of his period of study. It would practically separate the first from the second course students; in other words, the new beginners from the advanced scholars, and furnish a complete college term adapted to each. If a teacher in any of our ordinary institutions of learning should enter the school-room, and place the scholars just beginning to study arithmetic in the same class with those already advanced in the higher branches of mathematics, he would be declared *non compos mentis*, and speedily dismissed. And yet, under our present system of medical college instruction, we are continually placing the student who has scarcely become familiar with the outlines of the naked skeleton side by side with those who have already diligently pursued their studies three years, and compelling both to listen, day by day, to lectures on the same variety of subjects. By the proposed plan, each student would be obliged to become familiar with descriptive anatomy, histology, etc., before proceeding to surgical anatomy and practical surgery. So to, a full course on physiology, materia medica, and general pathology, would precede the study of practical medicine and obstetrics. It would thus not only render the system of medical study more methodical and rational, but the field actually covered by college instruction would be greatly enlarged. It is well known that under the present system, the professors of chemistry seldom progress beyond the elementary and inorganic part of their science before they are arrested by the *end* of the term. If the teachers of institutes, or physiology and general pathology, devote any adequate attention in the first part of the college term to histology and the important developments of the microscope, they almost necessarily arrive at the end before their course is half completed. If materia medica and medical jurisprudence are connected in the same chair, the latter is either not reached at all, or only three or four of its more prominent topics hastily presented during the last days of the term. The courses on the several practical branches are scarcely more complete. Thus, if the professor of surgery commences his course with due attention to surgical anatomy and pathology, he uniformly ends it with one-third of the important surgical diseases and accidents untouched.

But in the proposed plan, by increasing the whole number of professorships, and thereby securing a further division of labor, and by making the two terms consecutive and continuous instead of repetitional, these defects are obviated, and the important topics of histology, organic chemistry, toxicology, surgical anatomy, etc., not only have a name in the college curriculum, but actually receive the consideration which their importance demand. But one of the most important advantages of the proposed change relates to clinical teaching.

By restricting the number of lectures per day to five, sufficient time would be left to enable the student to devote one hour each day to attendance at the hospital; and if a proper understanding, or concert of action, existed between the hospital boards and the college faculties, it would be easy to so divide and classify the students that a limited number could visit some department of the hospitals each day. During the first term, their attention could be directed principally to the chemical and microscopic examination of morbid secretions, deposits, growths, etc.; to the individual practice of auscultation and percussion; and all those manipulations necessary to establish the habit of thoroughly investigating and correctly diagnosticating disease. Such a hospital course, in connection with the courses on physiology and general pathology in the college, would be of great advantage to the student, and properly prepare him for the details of therapeutics and operative surgery in the hospital during the second term in the college.

While the proposed system thus reduces the study of medicine to a systematic or methodical arrangement, practically enlarges the curriculum of college instruction to a very important degree, and renders more comprehensive and systematic the clinical teaching, it neither requires the student to remain materially longer in college at any one time, or to spend a larger aggregate amount of money during his period of study. The average aggregate length of the college terms under the present system does not exceed four months. This duplicated gives eight months as the aggregate time of college instruction required for obtaining a degree. While the system that we propose, instead of requiring a *duplicate* term of four months, exacts a primary term of four calendar months and a practical term of nearly five months, making an aggregate time of nearly nine months' attendance on the colleges.

But the increased expense required by the additional month would be offset by the less number of professors' tickets to be taken in each term or division of the collegiate year. This is an important item; for the fact cannot be denied, that nine-tenths of the medical students of our country are possessed of very limited pecuniary means. And whatever system of college instruction is devised, that does not regard this fact, will not only fail in practical efficiency, but will cause hundreds to enter upon the practice of medicine annually without any attendance upon college instruction during their pupilage.

Neither does the proposed plan *necessarily* require the schools to support a larger number of professors. The two annual terms being consecutive, and in some degree continuous, the same professor may properly and in some cases advantageously hold a chair in each term. This would be particularly the case with the departments of elementary chemistry in the first, and organic chemistry and toxicology in the second term. So also with general pathology in one, and practical medicine in the other; and descriptive anatomy, with regional and surgical anatomy. From much reflection during the last fifteen years, and nine years' active experience in teaching both in the college and the hospital, we are satisfied that the proposed plan will bear the most rigid scrutiny. And we hope the time has come when members of the profession, both in and out of the schools, will speak out freely, plainly and candidly on the whole subject.

